# Personality and Well-Being in Old Age

**DOI:** 10.1093/geroni/igaa057.1260

**Published:** 2020-12-16

**Authors:** Constanca Paul

**Affiliations:** University of Porto, Porto, Portugal

## Abstract

Personality is a controversial issues in Geropsychology. It is generally accepted that personality is relatively stable through life and plays a relevant role in life style and wellbeing. We assessed Personality with NEO (Neuroticism, Extroversion and Openness) in a community sample of 1322 individuals 55+ years, mean age 70.4 (sd=8.6) years, 71% women. The means were: Neuroticism 34.06(sd. 5.1); Extroversion 41.25(sd. 4.05), Openness 37.37(sd. 3.82), around 10 points higher than the normative Portuguese data. Considering the highest values of the 3th tertil in each domain, women have higher Neuroticism differing from men (p=0.001), and values raise with age along 3 age groups (p=0.001); Neuroticism has significant positive correlations (p<0.01) with loneliness, sleep problems, psychological distress, difficulties in ADL and IADL and self-perception of health, and negative correlations with social network, happiness and cognitive capacity. Extroversion do not differ between gender and varies by age group (p=0,04) in U inverted form. Extroversion is positively correlated with happiness and negatively with loneliness, self-perception of health, psychological distress, difficulties in ADL and IADL. Openness does not differ by gender but differ between age groups (p=0.18) in U shape and correlates negatively with cognitive capacity, and social network. Personality traits appear very relevant for successful aging as facilitators or risk factors of physical and mental health, and should be addressed to foster adaptation in old age.

